# Astragalus polysaccharide improve the proliferation and insulin secretion of mouse pancreatic β cells induced by high glucose and palmitic acid partially through promoting miR-136-5p and miR-149-5p expression

**DOI:** 10.1080/21655979.2021.1996314

**Published:** 2021-12-07

**Authors:** Shifang Deng, Lei Yang, Ke Ma, Wei Bian

**Affiliations:** a Department of Traditional Chinese Medicine, Shenzhen People’s Hospital (The Second Clinical Medical College, Jinan University; The First Affiliated Hospital, Southern University of Science and Technology), Shenzhen 518020, Guangdong, China; b Department of Geriatrics in Luohu Hospital of Traditional Chinese Medicine, Shenzhen Hospital of Shanghai University of traditional Chinese Medicine, Shenzhen, China

**Keywords:** Diabetes mellitus, Huang qi, MicroRNAs, Insulin-secreting Cells, Polysaccharides

## Abstract

The current drugs for the treatment of type 2 diabetes mellitus (T2DM) can cause side effects after long-time use. Hence, the novel drugs were urgent need to developed for T2DM patients. In this study, the effect of astragalus polysaccharide on dysfunctional insulin cells was investigated to clarify whether astragalus polysaccharide could be a novel drug for T2DM treatment. MIN6 cells (mouse pancreatic β-cell line) were treated with high glucose (HG)+ palmitic acid (PA) and then treated with astragalus polysaccharide. The proliferation, apoptosis, and insulin secretion were measured using CCK8, flow cytometry, and ELISA, respectively. Pancreatic and duodenal homeobox 1 (PDX1), miR-136-5p, and miR-149-5p expression levels were measured by RT-qPCR. The combination of EF-hand domain family member 2 (EFHD2) and miR-136-5p or miR-149-5p was analyzed by luciferase reporter assay. EFHD2 protein level was measured by western blot. We found that HG+PA treatment reduced MIN6 cell viability, insulin secretion, and PDX1 expression and promoted MIN6 cell apoptosis. Astragalus polysaccharide treatment reversed the effect of HG+PA on MIN6 cells. Additionally, astragalus polysaccharide treatment promoted miR-136-5p and miR-149-5p expression. Silencing of miR-136-5p and miR-149-5p expression partially reversed the therapeutic effects of astragalus polysaccharide. Furthermore, EFHD2 was the target of miR-136-5p and miR-149-5p. Meanwhile, astragalus polysaccharide treatment inhibited EFHD2 protein level in HG+PA treated MIN6 cell. Finally, EFHD2 overexpression partially reversed the therapeutic effects of astragalus polysaccharide. In conclusion, astragalus polysaccharide treatment improved proliferation and insulin secretion in HG+PA-treated MIN6 cells partially by promoting miR-136-5p and miR-149-5p expression to inhibit EFHD2 expression.

## Introduction

Type 2 diabetes mellitus (T2DM) is a multifactorial chronic disorder and accounts for the most prevalent type of patients with diabetes, approximately 90–95% [1,2]. Long-term hyperglycemia and high-saturated free fatty acids are the key contributors which inhibited insulin secretion, promoted pancreatic β-cell apoptosis, and decrease the number of normal functioning pancreatic β cells [3–5]. Therefore, maintaining pancreatic β-cell survival and improving functional pancreatic β-cells are the main therapeutic strategies for the clinical management of T2DM [6]. Various therapeutic agents, including sulfonylureas, α-glucosidase inhibitors, and biguanides have been used to treat T2DM, which can induce many side effects after long-term use [7]. Therefore, the novel drugs that are effective, nontoxic, and have a lighter burden for T2DM patients were urgent need to developed.

Previous studies have shown that biological macromolecules derived from natural products, mainly polysaccharides, are less toxic than chemically synthesized drugs and possess prominent efficacies on DM [8]. The dry root of *Astragalus membranaceus* (Chinese: Huang Qi) has been used as traditional Chinese medicine and health-care food [9]. Astragalus polysaccharide is the central active component which extracted from the stems or dried roots of *Astragalus membranaceus*. Previous studies have demonstrated that astragalus polysaccharide has anti-oxidative, anti-aging, anti-hypertensive, anti-tumor etc. pharmacological effects [10]. Moreover, it reduces blood sugar levels. Majorly, astragalus polysaccharide can effectively alleviate diabetes and diabetic complications such as diabetic foot, diabetic neuropathy, and diabetic cardiomyopathy [11]. However, the effect of astragalus polysaccharide on dysfunctional pancreatic β-cells and the underlying molecular mechanisms remain unclear.

MicroRNAs (miRNAs) can regulate cell proliferation, apoptosis, and inflammation, which are involved in the pathogenesis of DM [12–14]. However, whether astragalus polysaccharide can regulate dysregulated pancreatic β cells through miRNAs remains unclear.

In this study, we hypothesize that astragalus polysaccharide can improve insulin secretion in dysfunctional pancreatic β cells through miRNAs. First, MIN6 cells were induced by high glucose (HG) and palmitic acid (PA) to mimic glucolipotoxicity and establish the T2DM cell model [15]. Additionally, the effect of astragalus polysaccharide on HG+PA induced cells was investigated. Finally, the differential expression of miRNAs in T2DM and its role in astragalus polysaccharide treatment of MIN6 cells were studied. This study clarified the effect and mechanism of astragalus polysaccharide on glucolipotoxicity-induced mouse β-cell, which provides a theoretical basis for the clinical application of astragalus polysaccharide.

## Materials and methods

### Cell culture, treatments, and transfection

MIN6 cell was purchased from ZQXZbio (Shanghai, China) and cultured in RPMI-1640 with 10% FBS and 0.05 mM β-mercaptoethanol. To construct T2DM model *in vitro*, MIN6 cells were incubated in RPMI-1640 with 40 mM glucose and 0.4 mM PA with 0.5% BSA (model group) [15]. Astragalus polysaccharide (purity > 98%) was purchased from Macklin (No. A860847; lot: Shanghai, China). MIN6 cells in the model group were treated with 50, 100, and 200 μg/mL of astragalus polysaccharide (APS treatment group) or 100  μmol/L metformin (metformin group). MIN6 cells, which cultured under normal conditions, were used as a blank control group (blank group). The negative control inhibitor (NC inhibitor group), mmu-miR-136-5p, and mmu-miR-149-5p inhibitors (miR-136-5p inhibitor and miR-149-5p inhibitor groups) were purchased from Shanghai GenePharma (Shanghai, China). EF-hand domain family member 2 (EFHD2, NM_025994.3) cDNA include BamHI and EcoRI restriction enzyme cutting site was synthesized and directly jointed into pcDNA3.1 vector by Genewiz (Suzhou, China), which named EFHD2-OV. The pcDNA3.1 vector served as a negative control (NC-OV). A total of 3 μg EFHD2-OV and NC-OV and 100 nmol/L NC, mmu-miR-136-5p, and mmu-miR-149-5p inhibitors were transfected into the MIN6 cells, respectively, using Lipofectamine 2000 (Invitrogen; Thermo Fisher Scientific, Inc.) at 37°C for 24 h, then used for the subsequent experimentation.

### Cell proliferation and apoptosis assay

After astragalus polysaccharide treatment, the proliferation of MIN6 cells was measured using a Cell Counting Kit-8 (CCK-8, Dojindo, Kumamoto, Japan) at 450 nm [16]. MIN6 cell apoptosis was measured using the Annexin V-FITC Apoptosis Detection Kit (BD Bioscience, San Jose, CA, USA) using a BD FACSCanto Flow Cytometer (BD bioscience) [16].

### Glucose-stimulated insulin secretion (GSIS)

The assessment of GSIS was carried out as mentioned in previous studies [17]. Briefly, MIN6 cells were incubated in HEPES-balanced Krebs-Ringer bicarbonate buffer with 0.2% BSA (pH 7.4) for 2 h, then stimulated with low glucose (2.5 mmol/L) or high glucose (20 mmol/L) for 1 h. Insulin in the media was then quantified using a mouse insulin enzyme-linked immunosorbent assay (ELISA) Detection Kit (Sinobestbio, Shanghai, China). The insulin levels were then normalized based on the cell number.

### Real-time quantitative polymerase chain reaction assay

Pancreatic and duodenal homeobox 1 (PDX1) and miRNA expression levels were measured by real-time quantitative polymerase chain reaction (RT-qPCR). Total RNA extracted from MIN6 cells was used TRIzol (Invitrogen, Carlsbad, CA, USA) and then reverse-transcribed using the EasyScript First-Strand cDNA Synthesis SuperMix (TransGen Biotech, Beijing, China). RT-qPCR was performed with AceQ qPCR SYBR Green Master Mix (Vazyme) using the ABI PRISM® 7300 Sequence Detection System (Foster City, CA, USA). Relative expression levels were determined using the 2^−ΔΔct^ method [18]. Glyceraldehyde-3-phosphate dehydrogenase (GAPDH) and U6 were used as internal controls for PDX1 and miRNA, respectively. The primer sequences are shown as follow:

PDX1 forward primer (F): 5′-GAAATCCACCAAAGCTCACG-3′

PDX1 reverse primer (R): 5′-GTGTCTCTCGGTCAAGTTCA-3′

GAPDH F: 5′-GGCCTCCAAGGAGTAAGAAA-3′

GAPDH R: 5′-GCCCCTCCTGTTATTATGG-3′

mmu-miR-136-5p F: 5′-ACACTCCAGCTGGGACTCCATTTGTTTTGATGA-3′

mmu-miR-149-5p F: 5′-ACACTCCAGCTGGGTCTGGCTCCGTGTCTTCAC-3′

mmu-miR-374 c-5p F: 5′-ACACTCCAGCTGGGATAATACAACCTGCTAA-3′

mmu-miR-369-3p F: 5′-ACACTCCAGCTGGGAATAATACATGGTTGATC-3′

mmu-miR-411-5p F: 5′-ACACTCCAGCTGGGTAGTAGACCGTATAGCG-3′

mmu-miR-432 F: 5′-ACACTCCAGCTGGGTCTTGGAGTAGATCAGTGG-3′

Universal primer R: 5′-CTCAACTGGTGTCGTGGA-3′

U6-F: 5′-CTCGCTTCGGCAGCACA-3′

U6-R: 5′-AACGCTTCACGAATTTGCGT-3′

### Bioinformatics analysis and luciferase reporter assay

The potential target genes of miR-136-5p and miR-149-5p were analyzed using Starbase 3.0 [19]. Additionally, GSE20966 were chosen on Gene Expression Omnibus repository (GEO, https://www.ncbi.nlm.nih.gov/geo/) to analyzed the different-expressed mRNA in β cell between non-diabetic condition and diabetic condition [20]. GSE20966 dataset included 10 β cells from the pancreatic tissue sections of type 2 diabetic subjects (T2DM group) and 10 β cells from the pancreatic tissue sections of type 2 diabetic subjects (Normal group). Then, the differentially expressed mRNAs (DEmRNAs) were analyzed by GEO2R. The selection condition of DEmRNA is Padj<0.05 (T2DM group vs control group). In addition, wild-type EFHD2 (WT EFHD2) and mutated EFHD2 (Mut EFHD2) were inserted into psi-check2, which was co-transfected with miR-136-5p, miR-149-5p mimic, or NC mimic into 293 T using Lipofectamine 3000. At 24 h after co-transfection, luciferase assays were performed using the dual-luciferase reporter assay system (Promega), and the renilla/firefly luciferase activity ratio was calculated.

### Western blotting assay

EFHD2 protein level was measured by western blot and the operation steps refer to previous research [21]. Anti-EFHD2 antibody (ab106667, 1:1000 dilution), anti-GAPDH antibody (ab181602, 1:3000), and goat anti-rabbit IgG H&L (HRP) (ab205718, 1:10,000) were purchased from Abcam (Cambridge, MA, USA). The target proteins were visualized by chemiluminescence substrate (Keygentec) and the chemiluminescence signal was exposed to X-ray.

### Statistical analysis

All experiment was performed in triplicate. Normally distributed data is expressed as mean ± standard deviation and analyzed by the SPSS software (version 19.0; IBM, Chicago, IL, USA). The differences between the three groups were compared using one-way analysis of variance followed by Tukey’s post-hoc test. Statistical significance was set at P < 0.05.

## Results

### ASP improves survival and insulin secretion in HG+PA-treated MIN6 cells

**Figure 1.**
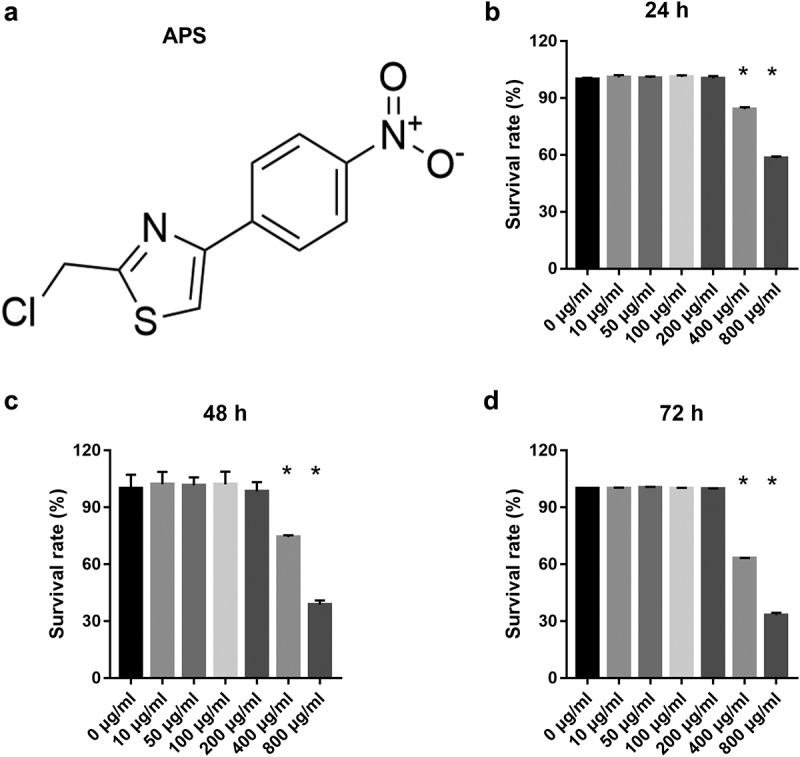
Effect of astragalus polysaccharide (APS) on the survival of MIN6 cells. (a) Molecular structural formula of astragalus polysaccharide. (b-d) The proliferation was measured by CCK8 after transfection at 24, 48, 72 h, then the survival rate was calculated according to the OD value of the NC inhibitor group. *P < 0.05, vs 0 μg/ml

**Figure 2.**
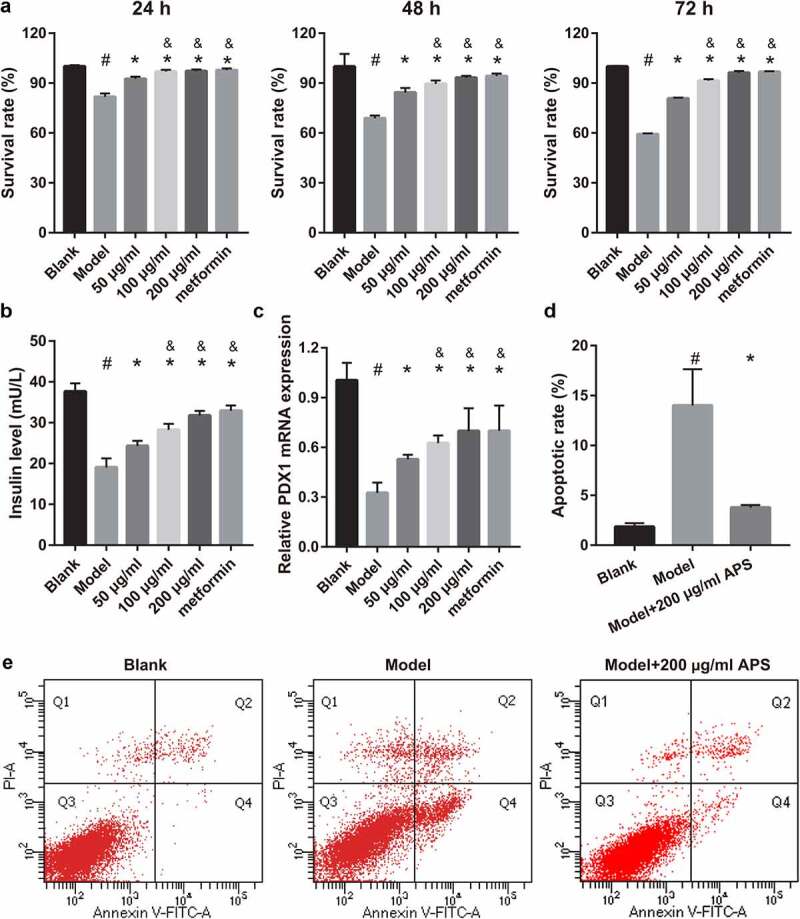
Astragalus polysaccharide (APS) promoted cell survival, insulin secretion, PDX1 mRNA expression and inhibited cell apoptosis in HG+PA-treated MIN6 cells. (a) Proliferation was measured by CCK8 after transfection at 24, 48, 72 h, then the survival rate was calculated according to the OD value of NC inhibitor group. (b) Insulin secretion was measured by ELISA after transfection at 48 h. (c) PDX1 mRNA expression was measured by RT-qPCR after transfection at 48 h. (d-e) Apoptosis was measured by flow cytometry after transfection at 48 h. ^#^P < 0.05, model group vs blank group; *P < 0.05, vs model group; ^&^P < 0.05, vs 50 μg/ml astragalus polysaccharide treatment group

To study the effect of astragalus polysaccharide on HG+PA-treated MIN6 cells, astragalus polysaccharide was added to treat HG+PA-treated MIN6 cells. The molecular structure of astragalus polysaccharide is shown in Figure 1(a). CCK8 results revealed that astragalus polysaccharide did not significantly inhibit cell viability when the astragalus polysaccharide concentration was less than 200 μg/mL (Figure 1(b-d)). When the astragalus polysaccharide concentration was more than 400 μg/mL, MIN6 cell viability was remarkably reduced after astragalus polysaccharide treatment for 24, 48, and 72 h (Figure 1(b-d)). Hence, 50, 100, and 200 μg/mL astragalus polysaccharide were selected as the low-, medium-, and high-dose groups for further study. MIN6 cell viability, insulin secretion, and PDX1 mRNA expression in the model group (HG+PA-treated) were remarkably decreased compared with the blank group (Figure 2(a-c)). After treatment, MIN6 cell viability, insulin secretion, and PDX1 mRNA expression in the 50, 100, and 200 μg/mL astragalus polysaccharide and metformin treatment groups were remarkably higher than those in the model group (Figure 2(a-c)). Compared with the 50 μg/mL astragalus polysaccharide treatment group, MIN6 cell viability, insulin secretion, and PDX1 mRNA expression in the 100 μg/mL and 200 μg/mL astragalus polysaccharide and metformin treatment groups significantly enhanced (Figure 2(a-c)). Additionally, MIN6 cell viability, insulin secretion, and PDX1 mRNA expression were not significantly different between the 100 μg/mL and 200 μg/mL astragalus polysaccharide and metformin treatment groups (Figure 2(a-c)). MIN6 cell apoptosis in the model group (HG+PA-treated) was remarkably more than that in the blank group, whereas MIN6 cell apoptosis was s remarkably reversed after 200 μg/mL astragalus polysaccharide treatment (Figure 2(d-e)).

### ASP promotes mmu-miR-136/149-5p expression in HG+PA-treated MIN6 cells

**Figure 3.**
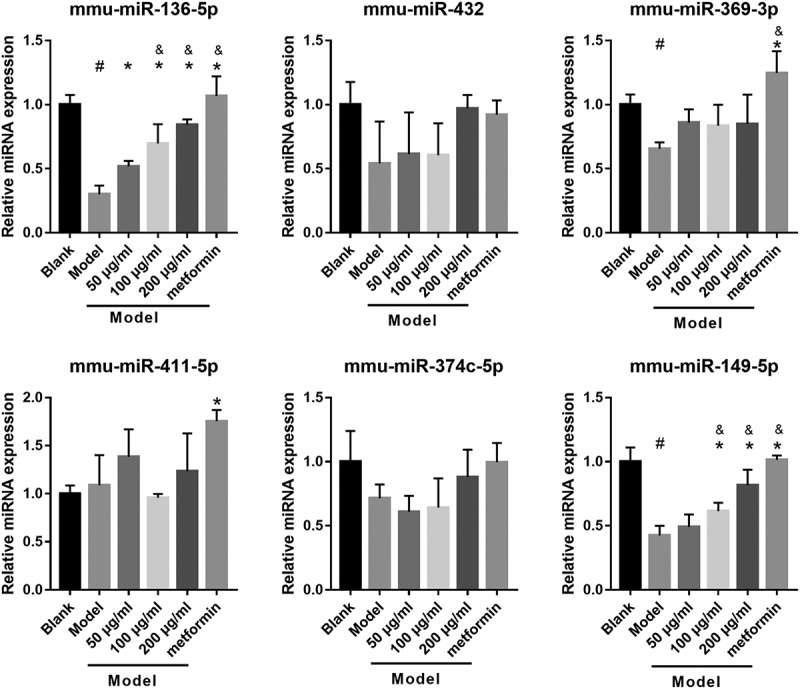
ASP treatment regulated miRNAs expression in HG+PA-treated MIN6 cells

To understand whether miRNA can participate in astragalus polysaccharide improving the insulin secretion of dysfunctional pancreatic β cells, this study analyzed the changes of the miRNAs expression in HG+PA-treated MIN6 cells under astragalus polysaccharide treatment. Previous studies have found that has-miR-136-5p (mmu-miR-136-5p), has-miR-149-5p (mmu-miR-149-5p), has-miR-369-3p (mmu-miR-369-3p), has-miR-411-5p (mmu-miR-411-5p), has-miR-432-5p (mmu-miR-432), and has-miR-655-3p (mmu-miR-374 c-5p) were significantly inhibited in T2DM islets according to ultra-high throughput sequencing and RT-qPCR results [22]. In this study, mmu-miR-374 c-5p, mmu-miR-411-5p, and mmu-miR-432 expression were not significantly different between the blank and model groups (Figure 3). Expression of mmu-miR-136-5p, mmu-miR-369-3p, and mmu-miR-149-5p in the model group were remarkably fewer than those in the blank group (Figure 3). In additional, mmu-miR-136-5p and mmu-miR-149-5p expression in the 100 μg/mL and 200 μg/mL astragalus polysaccharide and metformin treatment groups were remarkably more than those in the model group (Figure 3). Finally, compared with the 50 μg/mL astragalus polysaccharide treatment group, mmu-miR-136-5p and mmu-miR-149-5p expression in the 100 μg/mL and 200 μg/mL astragalus polysaccharide and metformin treatment groups significantly enhanced (Figure 3). These results suggested that only mmu-miR-136-5p and mmu-miR-149-5p expression changed in an astragalus polysaccharide concentration-dependent manner after astragalus polysaccharide treatment; hence, we chose mmu-miR-136-5p and mmu-miR-149-5p for further study.

Expression of miRNAs was measured by RT-qPCR after treatment at 48 h. ^#^P < 0.05, model group vs blank group; *P < 0.05, vs model group; ^&^P < 0.05, vs 50 μg/ml astragalus polysaccharide (APS) treatment group.

### Silencing miR-136-5p and miR-149-5p gene partially reverses the effect of astragalus polysaccharide on the HG+PA-treated MIN6 cells

**Figure 4.**
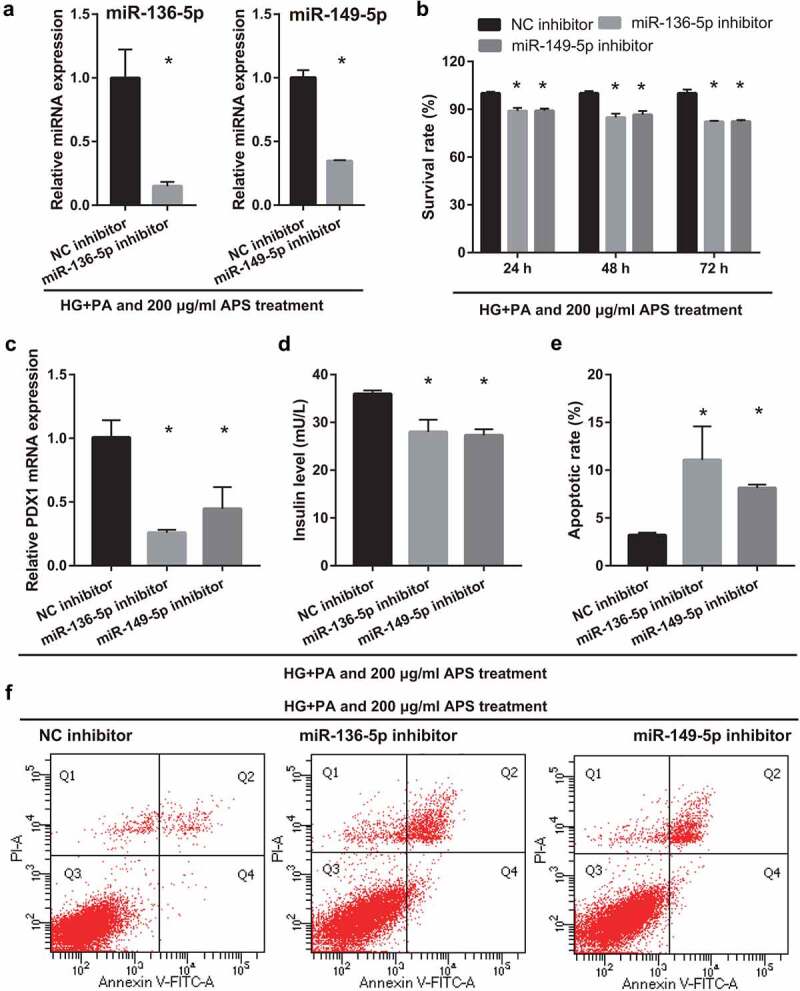
Silencing miR-136-5p and miR-149-5p gene reduced cell survival, insulin secretion, and PDX1 mRNA expression and promoted cell apoptosis in MIN6 cells co-treated with HG+PA and 200 μg/ml astragalus polysaccharide (APS). (a) Expression of miR-136-5p and miR-149-5p was measured by RT-qPCR after transfection at 24 h. (b) The proliferation was measured by CCK8 after transfection at 24, 48, 72 h, then the survival rate was calculated according to the OD value of NC inhibitor group. (c) PDX1 mRNA expression was measured by RT-qPCR after transfection at 48 h. (d) Insulin secretion was measured by ELISA after transfection at 48 h. (e-f) Apoptosis was measured by flow cytometry after transfection at 48 h. ^#^P < 0.05, vs NC inhibitor group

To understand whether mmu-miR-136-5p and mmu-miR-149-5p can participate in astragalus polysaccharide improved the insulin secretion of HG+PA-treated MIN6 cells, MIN6 cells were transfected with miR-136-5p and miR-149-5p inhibitor, respectively, and then co-treated with HG+PA and 200 μg/mL astragalus polysaccharide. After transfection for 24 h, miR-136-5p and miR-149-5p expression in miR-136-5p or miR-149-5p inhibitor groups were remarkably lower than that in the NC inhibitor group, respectively (Figure 4(a)). Additionally, MIN6 cell viability significantly reduced in miR-136-5p and miR-149-5p inhibitor groups after transfection at 24, 48, and 72 h compared with the NC inhibitor group (Figure 4(b)). And insulin secretion and PDX1 mRNA expression in MIN6 cells significantly reduced, whereas apoptosis increased in miR-136-5p and miR-149-5p inhibitor groups after transfection at 48 h (Figure 4(c-f)).

### Analysis of potential target genes of miR-136-5p and miR-149-5p

**Figure 5.**
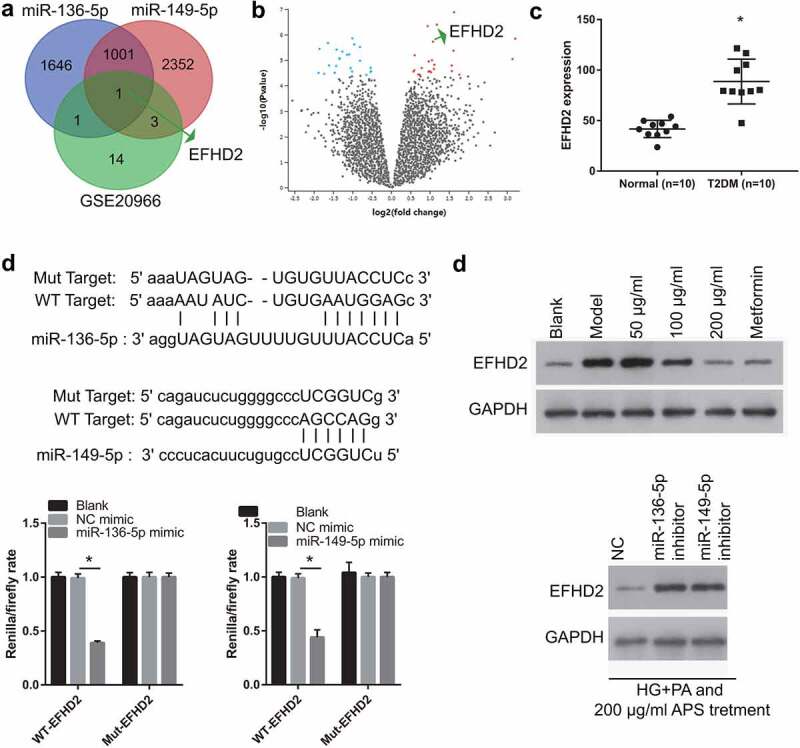
EFHD2 is the potential target genes of miR-136-5p and miR-149-5p. (a) The potential target genes of miR-136-5p and miR-149-5p were predicted by Starbase 3.0. And different-expressed mRNAs in β cell between non-diabetic condition and diabetic condition were analyzed by GEO2R in the GSE20966 dataset of Gene Expression Omnibus. Intersection of 3 sets of results was shown. (b) The heat map about the different-expressed mRNAs in β cell between non-diabetic condition and diabetic condition which analyzed by GEO2R in the GSE20966 dataset. Red: overexpression; Green: significant downexpression. (c) The EFHD2 expression was analyzed in GSE20966 dataset. (d) the bind site between miR-136-5p, miR-149-5p and 3′-UTR. And this bind was verified by luciferase reporter assay. The renilla/firefly ratio was significantly lower in miR-136-5p/miR-149-5p mimic + WT EFHD2 group than that in NC mimic + WT EFHD2 group, whereas the renilla/firefly ratio had no significantly changed between NC mimic + Mut EFHD2 group and miR-136-5p/miR-149-5p mimic + Mut EFHD2 group, suggesting that miR-136-5p and miR-149-5p can bind with the 3′-UTR of EFHD2. (e) EFHD2 expression was measured by western blot

To further understand the downstream genes regulated by mmu-miR-136-5p and mmu-miR-149-5p, this study used Starbase 3.0 to predict the target genes of mmu-miR-136-5p and mmu-miR-149-5p, and then the GSE20966 dataset was used to analyze the DEmRNAs in the pancreatic β cells of patients with T2DM, and the two groups were crossed to obtain the potential genes regulated by mmu-miR-136-5p and mmu-miR-149-5p. From the predictions of Starbase 3.0 and the results of GSE20966, it was only found one potential target gene of miR-136-5p and miR-149-5p (Figure 5(a)). The heat map of GSE20966 was shown in Figure 5(b). GSE20966 dataset found that 19 DEmRNAs were upregulated and 19 DEmRNAs were downregulated. Considering that miR-136-5p and miR-149-5p expression are down-regulated in HG+PA-induced MIN6 cells, this study only selected up-regulated DEmRNAs for follow-up studies. The result of GSE20966 showed that EFHD2 expression in the β cells of patient with diabetes mellitus was significantly higher than that in on-diabetes mellitus (Figure 5(c)). In addition, the binding site between miR-136-5p, miR-149-5p and 3′-UTR was shown in Figure 5(d). Moreover, compared with NC mimic + WT EFHD2 group, the renilla/firefly ratio was significantly inhibited in miR-136-5p/miR-149-5p mimic + WT EFHD2 group, whereas the renilla/firefly ratio had no significantly changed between NC mimic + Mut EFHD2 group and miR-136-5p/miR-149-5p mimic + Mut EFHD2 group, suggesting that miR-136-5p and miR-149-5p can bind with the 3′-UTR of EFHD2 (Figure 5(d)). Compared with blank group, EFHD2 expression was significantly promoted in model group. Whereas, its expression was significantly inhibited in HG+PA-treated MIN6 cells after astragalus polysaccharide and metformin treatment (Figure 5(e)).

### EFHD2 overexpression partially reverses the effect of astragalus polysaccharide on the HG+PA-treated MIN6 cells

**Figure 6.**
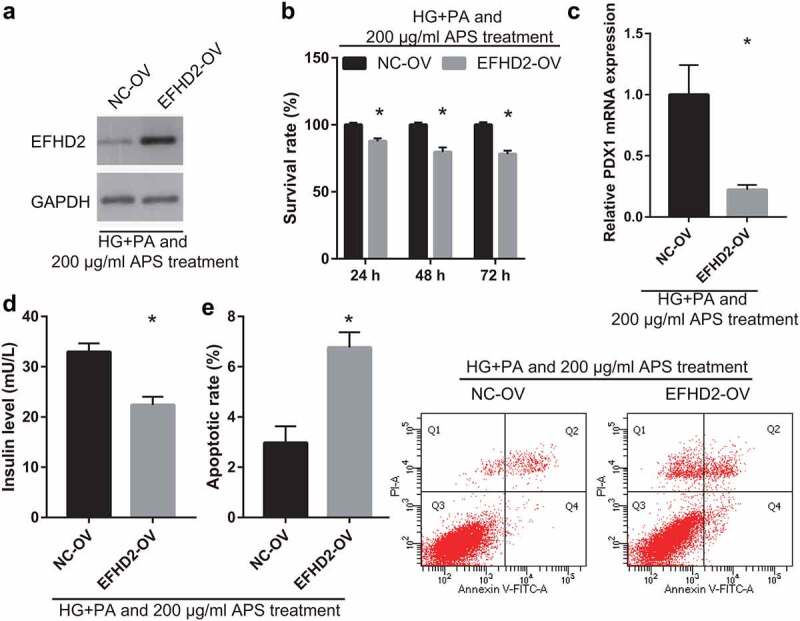
EFHD2 overexpression reverses the effect of astragalus polysaccharide (APS) on the HG+PA-treated MIN6 cells. In those experiments, MIN6 cells were transfected with EFHD2-OV or NC-OV and then co-treated with HG+PA and 200 μg/mL astragalus polysaccharide. (a) EFHD2 protein level, measured by western blot, was significantly increased after transfected EFHD2-OV at 48 h. (b) The survival rate was significantly reduced after transfected EFHD2-OV at 24, 48, 72 h. (c) PDX1 mRNA expression was measured by RT-qPCR after transfection at 48 h. (d) Insulin secretion was measured by ELISA after transfection at 48 h. (e) Apoptosis was measured by flow cytometry after transfection at 48 h. *P < 0.05, vs NC-OV group

To understand whether EFHD2 can participate in astragalus polysaccharide improving the insulin secretion of HG+PA-treated MIN6 cells, MIN6 cells were transfected with EFHD2-OV and then co-treated with HG+PA and 200 μg/mL astragalus polysaccharide. After transfection for 24 h, EFHD2 protein level in EFHD2-OV groups was remarkably more than that in the NC-OV group (Figure 6(a)). Additionally, compared with the NC-OV group, MIN6 cell viability significantly reduced in EFHD2-OV groups after transfection at 24, 48, and 72 h (Figure 6(b)). And insulin secretion and PDX1 mRNA expression in MIN6 cells significantly reduced, whereas apoptosis increased in EFHD2-OV groups after transfection at 48 h (Figure 6(c-e)).

### Discussion

The prevalence of T2DM is steadily increasing worldwide. This problem is compounded by the fact that current treatment drugs can lead to side effects after long-time use. Therefore, the novel, effective, and nontoxic drugs were urgent need to developed. In this study, we found that HG+PA treatment reduced insulin secretion and promoted MIN6 cell apoptosis. In contrast, the 200 μg/mL astragalus polysaccharide treatment reversed the effect of HG+PA treatment on MIN6 cells, playing a protective role. Astragalus polysaccharide may be a potential drug for the treatment of T2DM.

Astragalus polysaccharide is a monomer component extracted from the TCM of Huangqi (*Radix astragali mongolici*), which could improve plasma glucose, insulin resistance and secretion, and the complication of diabetes mellitus with promising effects in recent years [11]. However, the mechanism of action of astragalus polysaccharide on insulin cell apoptosis and secretion is still not well understood. With an increase in blood glucose levels and the continuous stimulation of hyperglycemia, the function and quality of pancreatic β cells reduced progressively, giving rise to abnormal insulin secretion in T2DM [23]. In this study, HG+PA was used to treat MIN6 cells to simulate the effect of the T2DM microenvironment on pancreatic β cells, and it was found that HG+PA treatment reduced MIN6 cell viability, insulin secretion, and PDX1 mRNA expression and promoted MIN6 cell apoptosis. PDX1 is a key transcriptional factor that maintains pancreatic β-cell survival and function, which is important for maintaining the stability of cell function and inhibiting the occurrence and development of diabetes [24]. Additionally, astragalus polysaccharide treatment, especially the 200 μg/mL astragalus polysaccharide treatment, reversed the effect of HG+PA treatment on MIN6 cells. There was no significant difference between the therapeutic effect of 200 μg/mL astragalus polysaccharide and the therapeutic effect of metformin. These results suggest that astragalus polysaccharide treatment attenuates HG+PA-induced apoptosis and abnormal insulin secretion.

Well-recognized mediators that maintain pancreatic β-cell survival and function, miRNAs, can become novel targets for the treatment of T2DM as well as potential diagnostic and prognostic markers [25,26]. In this study, astragalus polysaccharide treatment promoted miR-136-5p and miR-149-5p expression, reversed the effect of HG+PA treatment on miR-136-5p and miR-149-5p expression in MIN6 cells. miR-136-5p expression was inhibited in HG, PA, or inflammation-treated human β cell, which suggests that miR-136-5p can respond to high glucose, dyslipidemia, and inflammatory diabetic microenvironment [27]. Additionally, miR-136-5p improves blood glucose, urine protein concentrations, and renal fibrosis in diabetic rats [28]. A previous study found that miR-149-5p expression were remarkably decreased in interleukin-1β- and interferon-γ-treated EndoC-βH1 cells, which could enhance the expression of death protein 5, p53 up-regulated modulator of apoptosis, and Bcl2 associated X protein to increase apoptosis [29]. In human umbilical vein endothelial cells (HUVECs), miR-149-5p expression reduced after high glucose treatment [30]. Expression of miR-149-5p reduced in diabetic nephropathy, which promotes mesangial cell proliferation and fibrosis in mice [31]. miR-149-5p expression also reduced in high glucose-treated MIN6 cells, and the miR-149-5p mimic protected cell survival and insulin secretion while reduced cell apoptosis by inhibiting the expression of Bcl2-like 11 [6]. These studies suggest that miR-136-5p and miR-149-5p play the protective role in patients with diabetes and diabetic syndromes. Silencing miR-136-5p and miR-149-5p expression partially reversed the improve effect of astragalus polysaccharide. Similar with previous studies, the result suggested that miR-136-5p and miR-149-5p play the protective role in HG+PA-treated MIN6 cells. The result also suggested that astragalus polysaccharide treatment attenuates HG+PA-induced apoptosis and abnormal insulin secretion partially by promoting miR-136-5p and miR-149-5p expression.

EFHD2, also known as swiprosin-1, was first found in CD8+ lymphocytes [32]. Previous studies found that EFHD2 promote cell apoptosis in murine B cells and non-small cell lung cancer cells [33,34]. EFHD2 overexpression enhanced mitochondria-dependent apoptosis of glomerular podocytes while silenced EFHD2 reversed this effect in the early stage of mouse diabetic nephropathy [35,36]. In this study, astragalus polysaccharide treatment inhibited EFHD2 protein level, reversed the effect of HG+PA treatment on EFHD2 protein level in MIN6 cells. In addition, EFHD2 was the target gene of miR-136-5p and miR-149-5p in MIN6 cells. Finally, EFHD2 overexpression partially reversed the effect of astragalus polysaccharide in HG+PA-induced MIN6 cells. There results showed that astragalus polysaccharide treatment improved proliferation and insulin secretion in HG+PA-treated MIN6 cells partially by promoting miR-136-5p and miR-149-5p expression to inhibit EFHD2 expression.

However, there are certain limitations to the present study. The effect of astragalus polysaccharide treatment on pancreatic β-cells has not been verified in animals. Apart from EFHD2, mmu-miR-136-5p and mmu-miR-149-5p may have other regulatory targets in MIN6 cells. Furthermore, the downstream signaling pathways regulated by EFHD2 were need further verification. In addition, the functions of astragalus polysaccharide in patients with T2DM needs to be further studied.

## Conclusion

**Figure 7.**
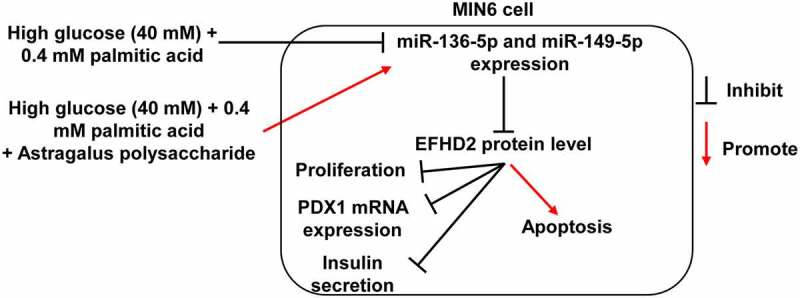
Mechanism diagram

HG+PA treatment reduced MIN6 cell viability, insulin secretion, and PDX1, miR-136-5p, and miR-149-5p expression and promoted MIN6 cell apoptosis. Astragalus polysaccharide treatment reversed the effect of HG+PA treatment on MIN6 cells partially by promoting miR-136-5p and miR-149-5p expression to inhibit EFHD2 expression. This study provides a new theoretical basis for the clinical application of astragalus polysaccharide (Figure 7).

Astragalus polysaccharide treatment promotes miR-136-5p and miR-149-5p expression and enhances the binding between miR-136-5p, miR-149-5p, and 3′-UTR EFHD2, then inhibits EFHD2 protein level to improve the proliferation and insulin secretion of pancreatic β cells inhibited by high glucose and palmitic acid.

## Data Availability

The original datasets are available from the corresponding author on reasonable request.

## Highlights

1. Astragalus polysaccharide improves the insulin secretion of MIN6 cell.
2. miR-136/149-5p is involved in the improvement effect of astragalus polysaccharide.
3. EFHD2 is the target gene of miR-136/149-5p in MIN6 cells.
4. EFHD2 is involved in the improvement effect of astragalus polysaccharide.
